# Genome-wide evolution and expression analysis of the *MYB-CC* gene family in *Brassica* spp.

**DOI:** 10.7717/peerj.12882

**Published:** 2022-02-25

**Authors:** Bin-Jie Gu, Yi-Kai Tong, You-Yi Wang, Mei-Li Zhang, Guang-Jing Ma, Xiao-Qin Wu, Jian-Feng Zhang, Fan Xu, Jun Li, Feng Ren

**Affiliations:** 1 Hubei Key Laboratory of Genetic Regulation and Integrative Biology, School of Life Sciences, Central China Normal University, Wuhan, Hubei, China; 2 Key Laboratory of Plant Germplasm Enhancement and Specialty Agriculture and Wuhan Botanical Garden, Chinese Academy of Sciences, Wuhan, Hubei, China; 3 Key Laboratory of Biology and Genetic Improvement of Oil Crops, Ministry of Agriculture, Oil Crops Research Institute of the Chinese Academy of Agricultural Sciences, Wuhan, Hubei, China

**Keywords:** MYB-CC, Evolution, *Brassica*, Diploid, Allotetraploid

## Abstract

The MYB-CC family is a subtype within the MYB superfamily. This family contains an MYB domain and a predicted coiled-coil (CC) domain. Several MYB-CC transcription factors are involved in the plant’s adaptability to low phosphate (Pi) stress. We identified 30, 34, and 55 *MYB-CC* genes in *Brassica rapa*, *Brassica oleracea*, and *Brassica napus*, respectively. The *MYB-CC* genes were divided into nine groups based on phylogenetic analysis. The analysis of the chromosome distribution and gene structure revealed that most *MYB-CC* genes retained the same relative position on the chromosomes and had similar gene structures during allotetraploidy. Evolutionary analysis showed that the ancestral whole-genome triplication (WGT) and the recent allopolyploidy are critical for the expansion of the *MYB-CC* gene family. The expression patterns of *MYB-CC* genes were found to be diverse in different tissues of the three *Brassica* species. Furthermore, the gene expression analysis under low Pi stress revealed that *MYB-CC* genes may be related to low Pi stress responses. These results may increase our understanding of *MYB-CC* gene family diversification and provide the basis for further analysis of the specific functions of *MYB-CC* genes in *Brassica* species.

## Introduction

Phosphorus (P) is an essential macronutrient for plant growth and development. It plays a vital role in a number of processes, including metabolic regulation, energy transfer, and protein activation (Marschner & Marschner, 2012). P is transported into the plant from the soil, which is key to maintaining P levels in plant cells (Raghothama & Karthikeyan, 2005). P is abundant in many soils, however, plants utilize a phosphate (Pi) form that is very scarce (Holford, 1997). Consequently, plants often encounter Pi deficiency in agricultural and natural systems’ soils (Raghothama, 1999). In order to improve the absorption and usage of Pi in the soils, plants have evolved many adaptive responses to low Pi stress, known as Pi starvation response (PSR) (Raghothama, 1999; Williamson et al., 2001; Vance, Uhde-Stone & Allan, 2003). Morphologically, the architecture of the root system is altered under low Pi stress, which leads to a high root-shoot ratio and the root hair proliferation to enhance the total surface area for soil exploration (Williamson et al., 2001; Lopez-Bucio et al., 2002). Plants increase the availability of endogenous and exogenous inorganic Pi by increasing the P-replacing enzyme activity in metabolites and structural compounds and by releasing organic acids into soil solution (Duff, Sarath & Plaxton, 1994; Raghothama, 1999; Vance, Uhde-Stone & Allan, 2003). Furthermore, the root tips of some plants combine with mycorrhizal fungi to form mutually beneficial symbionts to modify soil scavenging potential (Harrison, 1999; Watt & Evans, 1999).

In Arabidopsis’ PSR, a subset of genes is induced or suppressed (Wu et al., 2003; Misson et al., 2005). Pi starvation-induced (PSI) genes may be regulated by several transcription factors, of which PHOSPHATE STARVATION RESPONSE 1 (PHR1) is the key transcription factor (TF) in vascular plants (Rubio et al., 2001; Devaiah, Karthikeyan & Raghothama, 2007; Devaiah et al., 2009; Ren et al., 2012). In Arabidopsis, PHR1 with a MYB domain and a predicted coiled-coil (CC) domain belongs to a 15-member family of MYB-CC transcription factors (Rubio et al., 2001). PHR1 and its homologues are also members of GARP subfamily transcription factors as they contain a consensus sequence (SHLQ(K/M)(Y/F)) similar to the motif (SHAQK(Y/F)F) found in MYB-related proteins (Du et al., 2013; Safi et al., 2017). PHR1 may bind to the specific *cis*-element (PHR1-binding sequence, P1BS) with an imperfect palindromic sequence GNATATNC in the promoter of PSI genes (Rubio et al., 2001). The Arabidopsis *phr1* mutant displays a decreased expression of a subset of PSI genes and modified PSR, including a reduced Pi content and the accumulation of anthocyanin (Rubio et al., 2001; Misson et al., 2005). However, the overexpression of *PHR1* in transgenic Arabidopsis increased the expression of a subset of PSI genes, the Pi content, and anthocyanin accumulation (Nilsson, Muller & Nielsen, 2007). The binding of PHR1 to P1BS elements in PSI gene promoters may be modulated by the SPX domain-containing proteins, such as SPX1 and SPX2, which interact with PHR1 and its orthologs to inhibit their activity, thereby suppressing PSR (Puga et al., 2014; Wang et al., 2014; Wild et al., 2016). PHR1 and the other MYB-CC members (PHL1, PHL2, PHL3 and PHL4) are crucial components of the central regulatory system controlling Arabidopsis’ transcriptional responses to Pi starvation (Bustos et al., 2010; Sun et al., 2016; Wang et al., 2018). Several *PHR1* ortholog genes have been identified and characterized in different species (Valdes-Lopez et al., 2008; Zhou et al., 2008; Ren et al., 2012; Wang et al., 2013; Wang et al., 2019). The identification of PHR1 homologs and the establishment of their roles in these species have shown that central regulators have highly conserved functions. Furthermore, it suggests that members of the MYB-CC family may play a transcriptional regulatory role in controlling Pi uptake and homeostasis in plants. Therefore, it is crucial to identify *MYB-CC* genes and explore their regulatory roles in plant Pi uptake and homeostasis.

*Brassica napus* is a global essential oil crop. It is primarily cultivated for its healthy edible oil that is extracted from the seeds and its utility as a renewable biofuel, which is receiving increased attention. Vegetable types of the species have also been bred for consumption by both human and animals. *B. napus* is an allopolyploid (AACC, 2n = 38) resulting from natural interspecific hybridization between its two parental species: *Brassica rapa* (AA, 2n = 20) and *Brassica oleracea* (CC, 2n = 18) (Beilstein, Al-Shehbaz & Kellogg, 2006; Chalhoub et al., 2014). The genome evolution assay indicates the whole-genome triplication event in the *Brassica* lineage occurred about 13–17 million years ago (Ma) after it diverged from *Arabidopsis thaliana* about 20 Ma (Johnston et al., 2005; Town et al., 2006; Yang et al., 2006; Lysak et al., 2007; Mun et al., 2009). *B. oleracea* and *B. rapa* are also important vegetable crop species with a wide genetic and morphological diversity. *B. rapa* and *B. oleracea* differentiated from their ancestor approximately 3.75 Ma (Inaba & Nishio, 2002). The genome was doubled to form the heterotetraploid *B. napus* about 10,000 years ago (Cheung et al., 2009). Homologous fragment sequences in *B. napus*, *B. rapa*, and *B. oleracea* genomes indicate that there is a near-perfect microcollinearity between them (Cheung et al., 2009; Wang et al., 2011).

Our previous study revealed that *BnPHR1* may play a crucial regulatory role in the Pi starvation response in *B. napus* (Ren et al., 2012). However, there is still a lack of research on other *MYB-CC* genes in *B. napus* and its parental species, *B. rapa* and *B. oleracea*. We conducted an evolutionary-based genome-wide analysis of the *MYB-CC* gene family in *Brassica* species. The gene structure, chromosome distribution, conserved motifs, phylogenetic relationship, *cis-*elements, and expression profiles were assayed systematically. These results may illuminate the evolutionary history of the *MYB-CC* gene family and assist in the investigation of the functions of *MYB-CC* genes in the *Brassica* species.

## Materials and Methods

### Plant materials and growth conditions

*B. napus* (accession Zhongshuang11) was kindly provided by the Oilcrops Research Institute, Chinese Academy of Agricultural Sciences. The seeds were disinfected in 70% ethanol for 1 min, washed with ddH_2_O three to four times, placed on a Petri dish lined with two layers of filter paper, and then germinated at 24 °C in growth chamber. After 3 days, the germinated seedlings were transferred to MS medium containing 0.3% agar and 3% sucrose at pH 5.7. The seedlings grew at 24 °C under a long-day light cycle (16 h light/8 h dark) and a relative humidity of 65%. Then the two-week-old plants were cultured in 1 mM and 1 μM Pi liquid medium for 24 h, respectively. Rapeseed seedlings’ shoots and roots were collected and frozen in liquid nitrogen. The RNA-seq analysis was carried out by Biomarker (Beijing). A total of 16 RNA samples were sequenced using the Illumina HiSeq 2,000 platform (Illumina, USA). The transcript abundance (FPKM value) was calculated based on the length of the gene and the reads mapped gene. The heatmap was constructed using TBtools (Chen et al., 2020). The raw RNA-seq data were deposited in the NCBI SRA database (https://ncbi.nlm.nih.gov/sra/) under the accession number PRJNA739537 and the assembled transcriptome data were deposited in the NCBI GEO database (https://www.ncbi.nlm.nih.gov/geo/)under the accession number GSE192452.

### Identification of MYB-CC gene family in *B. napus, B. rapa*, and *B. oleracea*

The genes and proteins sequences in *B. napus*, *B. rapa*, and *B. oleracea* were downloaded from two databases (Genoscope: https://www.genoscope.cns.fr/brassicanapus/ and BRAD: http://brassicadb.cn/). The *MYB-CC* members were selected from *B. napus*, *B. rapa*, and *B. oleracea* by considering the E value < e^−10^ when compared with the 15 known *MYB-CC* protein sequences from *A. thaliana* (TAIR: http://www.arabidopsis.org/). This was achieved using BLAST on a computer (Rubio et al., 2001). The candidate protein sequences were further filtered using PFAM (http://pfam.xfam.org/) (Finn et al., 2014) and InterProScan (http://www.ebi.ac.uk/interpro) (Mitchell et al., 2015) according to the MYB and CC domains. The molecular weight and isoelectric point of MYB-CC members were calculated with ExPASy (Gasteiger et al., 2003).

### Multiple sequence alignment and phylogenetic analysis of MYB-CC family

We constructed multiple sequence alignments of BnaMYB-CC, BraMYB-CC, BolMYB-CC, and AtMYB-CC using Clustal X (Thompson et al., 1997). The unrooted phylogenetic trees were constructed using the Neighbor-Joining (NJ) method. This was done using MEGA 6.0 software with the JTT model and pairwise gap deletion option (Tamura et al., 2013). The bootstrap analysis was conducted with 1,000 iterations.

### Chromosomal location and gene duplication

The position of the *MYB-CC* genes was investigated according to the *B. napus* v4.1.chromosomal database (http://www.genoscope.cns.fr) and the Brassica database (BRAD, http://brassicadb.org). MapInspect software was used to locate the *MYB-CC* genes. The gene duplication was defined as: a coverage region between two sequences of more than 70% with more than 85% similarity in the coverage region (Zhou et al., 2004; Yang et al., 2008). Additionally, the *ka* (non-synonymous mutation rate) and *ks* (synonymous mutation rate) values of the repeating gene pairs were calculated using DnaSp software.

### Gene structure and conserved motif analysis

GSDS (http://gsds.gao-lab.org/) was used to determine the exon/intron distribution of *BnaMYB-CCs* through the comparison of CDS and genomic sequences (Guo et al., 2007). The conserved motifs of BnaMYB-CCs were identified using MEME (Bailey et al., 2006) with the flowing parameters: the maximum number of motif: 10; the optimum width: 6–250; the number of repetitions: any. InterProScan was used to annotate the motifs (Quevillon et al., 2005).

### Promoter elements analysis

From BRAD, a sequence of 2,000 bp upstream of the start codon was obtained for each *MYB-CC* gene and submitted to the PlantCARE website to determine the distribution of *cis*-elements in *MYB-CC* promoters (http://bioinformatics.psb.ugent.be/webtools/plantcare/html/) (Lescot et al., 2002).

### Gene expression patterns in different tissues

The expression patterns of *MYB-CC* genes in different *B. rapa, B. oleracea*, and *B. napus* tissues were analyzed with previously published transcriptomic data (Tong et al., 2013; Yu et al., 2014; Sun et al., 2017). RNA-seq data were collected from the GEO database using accession numbers GSE43245 and GSE42891.

## Results

### Identification of MYB-CC genes in *B. napus, B. rapa*, and *B. oleracea*

The *B. napus*, *B. rapa*, and *B. oleracea* genomes were searched using the protein sequences of 15 MYB-CCs in *A. thaliana*. A total of 55 *MYB-CC* genes were identified in *B. napus* and were equally distributed in the A_n_ and C_n_ subgenomes (27 genes in A_n_ and 28 genes in C_n_, respectively) (Table 1). A nomenclature system was used to distinguish the *MYB-CC* genes. The identified *MYB-CC* genes in *B. napus* were designated as *BnaMYB-CC01* to *BnaMYB-CC55* based on their order on A_n_01, C_n_01, A_n_02, C_n_02, etc.. The majority of BnaMYB-CCs contained 148 to 526 amino acids with molecular weights ranging from 16.991 kDa to 59.691 kDa. The predicted isoelectric points (pI) varied from 4.99 to 10.55 (Table 1). A total of 30 *MYB-CC* genes were identified in *B. rapa* and were designated as *BraMYB-CC01* to *BraMYB-CC30* based on their order on A_r_01-Ann (Table S1). Furthermore, 34 *MYB-CC* genes were identified in *B. oleracea* and were assigned as *BolMYB-CC01* to *BolMYB-CC34* based on their order on C_o_01-Cnn (Table S2).

**Table 1:** MYB-CC gene family in *B. napus*.

Gene name	Gene symbol	Length (aa)	MW (Da)	pI	chr.Location
*BnaMYB-CC01*	*BnaA01g37390D*	384	43,238.60	6.96	chrA01_random:2673192..2675057
*BnaMYB-CC02*	*BnaA01g08300D*	417	45,819.52	6.08	chrA01:3948585..3951254
*BnaMYB-CC03*	*BnaA01g23340D*	292	31,712.71	6.24	chrA01:15684038..15686754
*BnaMYB-CC04*	*BnaA01g30110D*	437	48,643.16	5.75	chrA01:20712451..20715282
*BnaMYB-CC05*	*BnaA01g30340D*	234	26,916.35	6.90	chrA01:20844513..20846132
*BnaMYB-CC06*	*BnaC01g09850D*	403	44,256.62	6.03	chrC01:5804612..5807008
*BnaMYB-CC07*	*BnaC01g30360D*	292	31,703.72	6.24	chrC01:28760606..28763683
*BnaMYB-CC08*	*BnaC01g38060D*	437	48,633.04	5.63	chrC01:37146750..37149877
*BnaMYB-CC09*	*BnaA02g36130D*	148	16,991.72	9.67	chrA02_random:724386..725308
*BnaMYB-CC10*	*BnaA02g14410D*	324	36,771.36	6.29	chrA02:8128787..8130586
*BnaMYB-CC11*	*BnaA02g30980D*	401	44,544.40	4.99	chrA02:22387811..22390602
*BnaMYB-CC12*	*BnaC02g01850D*	376	42,385.10	6.72	chrC02:789792..792229
*BnaMYB-CC13*	*BnaC02g19330D*	334	37,761.68	7.13	chrC02:15635506..15637277
*BnaMYB-CC14*	*BnaC02g39290D*	314	35,590.55	5.07	chrC02:42253955..42255665
*BnaMYB-CC15*	*BnaA03g55680D*	372	42,063.01	7.20	chrA03_random:375685..378053
*BnaMYB-CC16*	*BnaA03g32590D*	432	48,203.65	5.71	chrA03:15741533..15744207
*BnaMYB-CC17*	*BnaA03g37160D*	294	32,195.22	6.20	chrA03:18367120..18369001
*BnaMYB-CC18*	*BnaC03g73670D*	422	47,150.68	5.63	chrC03_random:1699065..1702064
*BnaMYB-CC19*	*BnaC03g43430D*	294	32,237.26	6.41	chrC03:28447863..28449907
*BnaMYB-CC20*	*BnaA05g37380D*	381	42,773.13	6.37	chrA05_random:2969117..2971012
*BnaMYB-CC21*	*BnaA05g33010D*	352	39,166.69	5.61	chrA05:22501618..22504921
*BnaMYB-CC22*	*BnaC05g40460D*	231	26,542.02	7.23	chrC05:38626692..38627882
*BnaMYB-CC23*	*BnaC05g47270D*	397	44,239.56	5.60	chrC05:42338193..42340293
*BnaMYB-CC24*	*BnaC05g47520D*	372	41,833.12	6.51	chrC05:42526968..42528869
*BnaMYB-CC25*	*BnaA06g00030D*	364	40,365.02	5.92	chrA06:15215..16810
*BnaMYB-CC26*	*BnaC06g19840D*	343	38,387.30	8.76	chrC06:21970139..21972032
*BnaMYB-CC27*	*BnaC06g25290D*	352	40,134.09	5.65	chrC06:26862555..26864876
*BnaMYB-CC28*	*BnaC06g28050D*	334	37,763.62	7.12	chrC06:29356276..29358118
*BnaMYB-CC29*	*BnaC06g39850D*	355	39,560.42	8.40	chrC06:36783795..36785668
*BnaMYB-CC30*	*BnaA07g00280D*	338	37,838.63	6.01	chrA07:257201..258856
*BnaMYB-CC31*	*BnaA07g05970D*	212	24,849.54	10.55	chrA07:6257304..6259312
*BnaMYB-CC32*	*BnaA07g20350D*	343	38,340.27	8.76	chrA07:15976488..15978194
*BnaMYB-CC33*	*BnaA07g24220D*	321	36,838.51	6.00	chrA07:18088750..18090258
*BnaMYB-CC34*	*BnaA07g28130D*	331	37,499.33	7.12	chrA07:20352170..20354016
*BnaMYB-CC35*	*BnaA07g34920D*	355	39,531.42	8.40	chrA07:23644702..23646584
*BnaMYB-CC36*	*BnaC07g00540D*	340	38,246.02	5.67	chrC07:701526..703146
*BnaMYB-CC37*	*BnaC07g07340D*	526	59,691.65	7.12	chrC07:11638655..11641909
*BnaMYB-CC38*	*BnaC07g18520D*	280	31,234.19	7.72	chrC07:25179396..25180928
*BnaMYB-CC39*	*BnaC07g41450D*	422	46,905.98	6.42	chrC07:41325045..41327724
*BnaMYB-CC40*	*BnaA08g13620D*	396	43,731.26	5.75	chrA08:11763924..11766763
*BnaMYB-CC41*	*BnaC08g08750D*	282	31,016.14	7.09	chrC08:13171439..13172925
*BnaMYB-CC42*	*BnaC08g13160D*	398	43,966.59	5.81	chrC08:18195906..18198659
*BnaMYB-CC43*	*BnaA09g03540D*	409	45,786.78	6.16	chrA09:1797716..1800204
*BnaMYB-CC44*	*BnaA09g10270D*	317	35,908.38	5.13	chrA09:5256551..5258734
*BnaMYB-CC45*	*BnaC09g02840D*	411	45,904.72	5.73	chrC09:1647123..1648856
*BnaMYB-CC46*	*BnaC09g54190D*	393	44,222.36	6.00	chrC09_random:4002389..4004564
*BnaMYB-CC47*	*BnaC09g10430D*	326	36,745.23	5.43	chrC09:7051680..7053999
*BnaMYB-CC48*	*BnaC09g48760D*	335	37,665.00	8.33	chrC09:47473893..47476388
*BnaMYB-CC49*	*BnaA10g16590D*	392	44,300.48	5.91	chrA10:12579079..12581358
*BnaMYB-CC50*	*BnaA10g24150D*	374	42,216.30	7.66	chrA10:15810902..15813379
*BnaMYB-CC51*	*BnaAnng01860D*	375	42,283.01	6.69	chrAnn
*BnaMYB-CC52*	*BnaAnng05640D*	280	31,236.12	8.54	chrAnn
*BnaMYB-CC53*	*BnaCnng44310D*	372	42,127.01	7.20	chrCnn
*BnaMYB-CC54*	*BnaCnng52990D*	381	43,430.00	8.56	chrCnn
*BnaMYB-CC55*	*BnaCnng55690D*	353	39,424.12	6.04	chrCnn

### Evolutionary analysis of MYB-CC family

**Figure 1:**
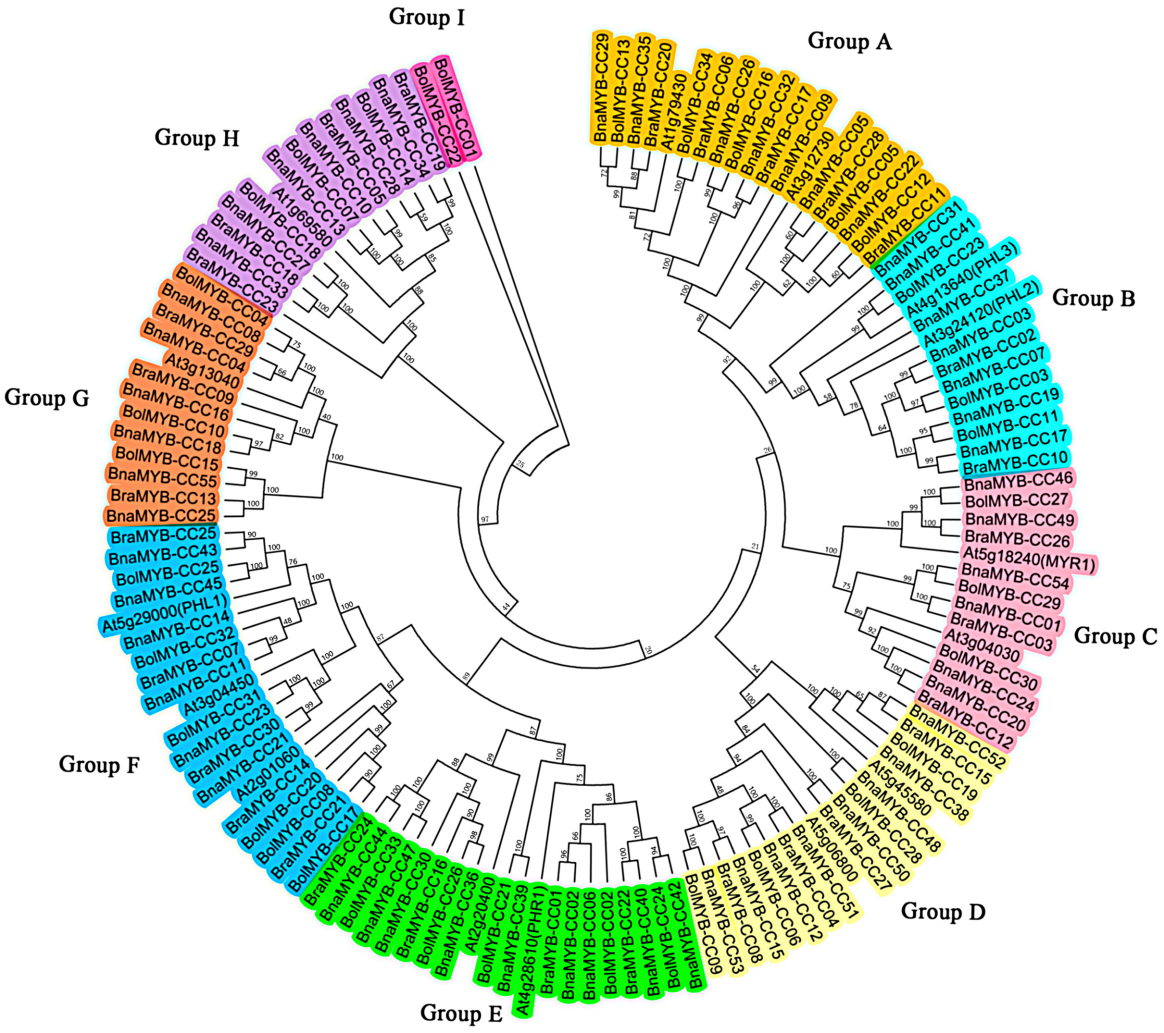
Phylogenetic tree of MYB-CC proteins of *A. thaliana*, *B. napus*, *B. rapa*, and *B. oleracea*. The phylogenetic tree was generated using the Neighbor-Joining (NJ) method implemented in the MEGA 6.0 software with JTT model and pairwise gap deletion option. The bootstrap analysis was conducted with 1,000 iterations.

To explore the evolutionary relationship of the MYB-CC gene family, an unrooted phylogenetic tree was generated using the Neiboring-Joining (NJ) method based on the full-length of 134 MYB-CC protein sequences from *A. thaliana, B. napus, B. rapa*, and *B. oleracea*. As shown in Fig. 1, the MYB-CC proteins were classified into nine distinct groups based on the branch of the phylogenetic tree. Group E and Group F each had 20 members, which were the larger groups. Group I was the smallest group with only two MYB-CC members. Group A, B, C, D, G, and H contained 19, 14, 14, 16, 13, and 14 members, respectively. One of the Arabidopsis MYB-CC members (coded by At2g01060) was not found to have a homolog in *B. napus*. The MYB-CC proteins in Group E were homologous to AtPHR1 and AtPHL4, and the members of Group B were homologs of AtPHL2 and AtPHL3. AtPHL1 belonged to Group F (Fig. 1).

### Chromosomal distribution of MYB-CC genes

**Figure 2:**
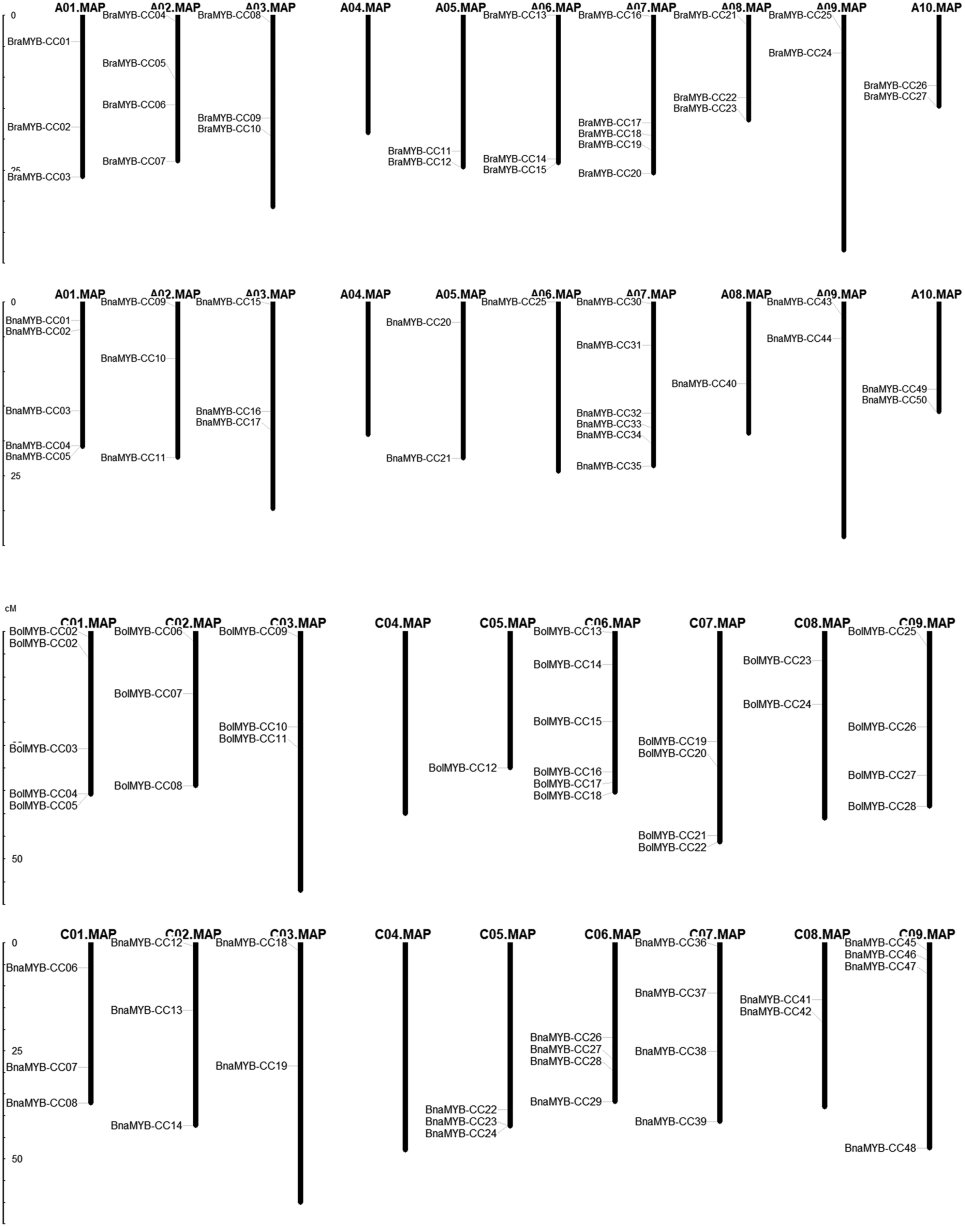
Distribution of *MYB-CC* genes in genomes of *B. napus*, *B. rapa*, and *B. oleracea*.

In order to determine the chromosomal distribution of *MYB-CC* genes, we searched the *Brassica* genome database and mapped *MYB-CC* genes to the corresponding chromosomes. The results showed that 50 *BnaMYB-CC* genes were distributed across 17 chromosomes except for A_n_04 and C_n_04 (Fig. 2). There were 27 *BraMYB-CC* genes located on nine chromosomes except for A_r_04, and 28 *BolMYB-CC* genes were distributed on eight chromosomes except for C_o_04 (Fig. 2). Five *BnaMYB-CC* genes (*BnaMYB-CC51*, *BnaMYB-CC52*, *BnaMYB-CC53*, *BnaMYB-CC54*, and *BnaMYB-CC55*), three *BraMYB-CC* genes (*BraMYB-CC28*, *BraMYB-CC29*, and *BraMYB-CC30*), and six *BolMYB-CC* genes (*BolMYB-CC29*, *BolMYB-CC30*, *BolMYB-CC31*, *BolMYB-CC32*, *BolMYB-CC33*, and *BolMYB-CC34*) were mapped onto unanchored scaffolds (http://www.genoscope.cns.fr and http://brassicadb.org). In the A_n_ subgenome of *B. napus*, chromosome A_n_07 carried the most (seven) *MYB-CC* genes. Chromosome A_n_06 and A_n_08 only contained one *MYB-CC* gene and chromosome A_n_04 did not have *MYB-CC* genes. Comparatively, *BnaMYB-CC* genes were more prevalent in the C_n_ subgenome. Except for chromosome C_n_04, the other chromosomes in the C_n_ subgenome contained two to four *MYB-CC* genes (Table S3).

The relative position of seven *MYB-CC* genes changed on the chromosomes after allotetraploidy. By evolving from diploid to allotetraploid, two *BraMYB-CC* genes were rearranged in the A_n_ subgenome belonging to *B. napus*. Comparatively, the positions of five *MYB-CC* genes in the C_o_ and C_n_ were changed, of which three genes were on chromosome C06 (Fig. 2; Table S4). *MYB-CC* genes were located on several chromosomes, namely A_r_03-A_n_03, A_r_09-A_n_09, A_r_10-A_n_10, and C_o_08-C_n_08. This was a conservative process compared to the gene distribution of *B. napus* and its diploid ancestors (Fig. 2). The results indicated that the genetic variation took place during the evolution of the *B. napus* genome from its diploid progenitors. The A_n_ subgenome was more stable than the C_n_ subgenome, which may be due to the more abundant homologous exchanges or richer transposable elements in the C subgenome (Chalhoub et al., 2014).

### Collinearity analysis and gene duplication

**Figure 3:**
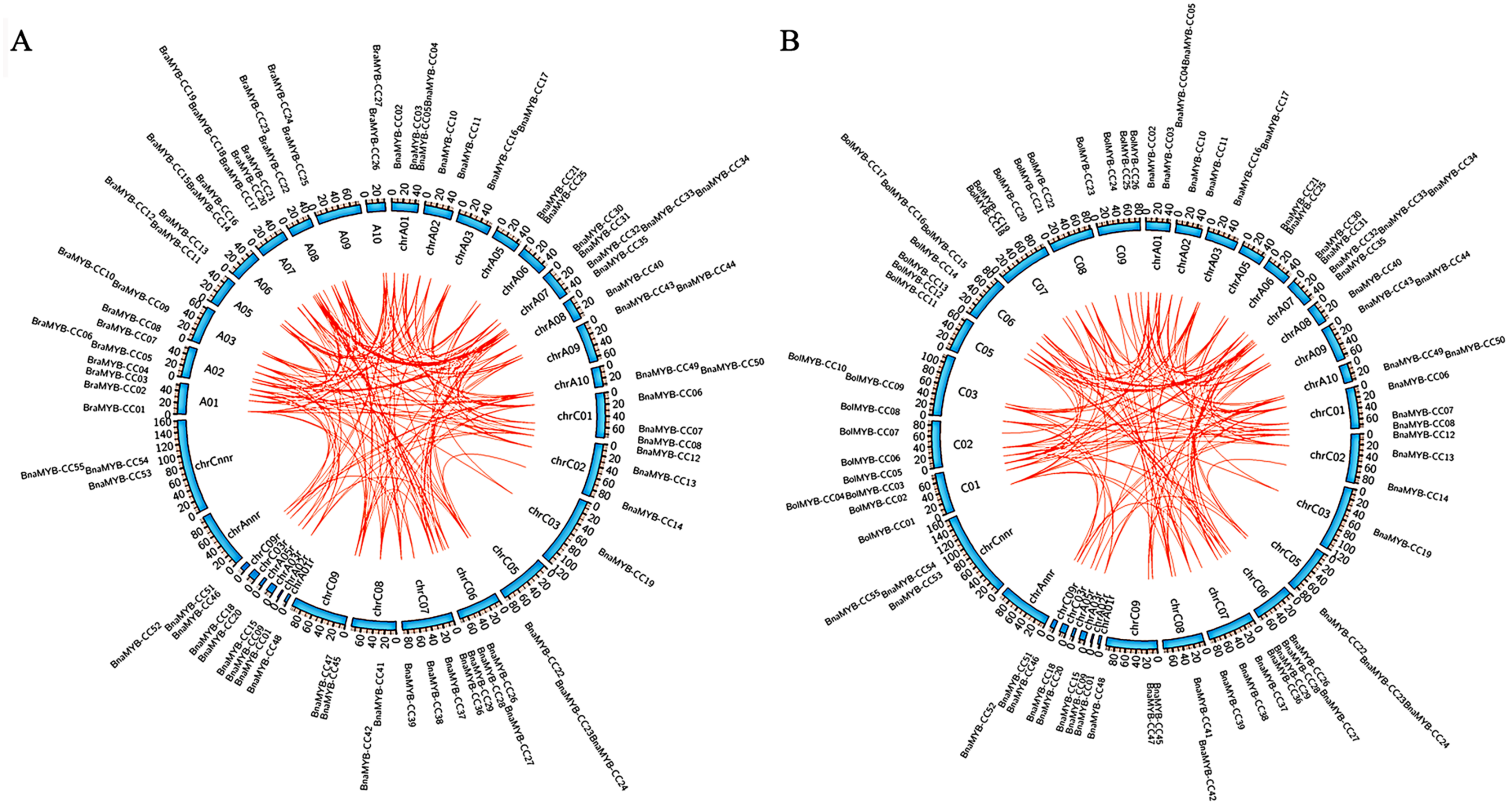
Collinearity analysis of *MYB-CC* genes of *B. napus*, *B. rapa*, and *B. oleracea*.

Collinearity analysis of the *MYB-CC* genes was performed on *B. napus* and its two parental species (*B. rapa* and *B. oleracea*). As shown in Fig. 3, the collinear *MYB-CC* genes were widely distributed in the two subgenomes (A_n_ and C_n_) and two genomes (A_r_ and C_o_), indicating that they promote evolution in the *MYB-CC* gene family. We also revealed the extension mechanism of the *MYB-CC* gene family in *B. napus* by studying gene duplication events. The results showed that 35 *BnaMYB-CC* genes formed 36 gene pairs with a high homology between gene and protein sequences. Some genes were involved in the duplication events more than once (Table 2). Among these segmental duplication events, 34 events happened between diverse chromosomes, and two events occurred on the same chromosome (*BnaMYB-CC26* and *BnaMYB-CC29* on C_n_06; *BnaMYB-CC32* and *BnaMYB-CC35* on A_n_07) (Fig. 2). Tandem duplication genes were described as a cluster of duplicated genes within 200 kb (Zhu et al., 2020). However, the two pairs of duplicated genes on the same chromosome were not closely related (Fig. 2; Table 1), suggesting that they were not the product of tandem duplication events.

**Table 2:** *ka* and *ks* of duplicated gene pairs.

Gene pairs		*ks*	*ka*	*ka/ks*
*BnaMYB-CC01*	*BnaMYB-CC20*	0.2596	0.0519	0.1999
*BnaMYB-CC01*	*BnaMYB-CC24*	0.2514	0.0386	0.1535
*BnaMYB-CC02*	*BnaMYB-CC06*	2.2654	2.7285	1.2044
*BnaMYB-CC02*	*BnaMYB-CC40*	0.3314	0.0599	0.1807
*BnaMYB-CC02*	*BnaMYB-CC42*	0.3707	0.0710	0.1915
*BnaMYB-CC03*	*BnaMYB-CC07*	0.0785	0.0091	0.1159
*BnaMYB-CC03*	*BnaMYB-CC17*	0.3526	0.0588	0.1668
*BnaMYB-CC03*	*BnaMYB-CC19*	0.3526	0.0588	0.1668
*BnaMYB-CC04*	*BnaMYB-CC08*	0.0986	0.0222	0.2252
*BnaMYB-CC06*	*BnaMYB-CC40*	0.3713	0.0689	0.1856
*BnaMYB-CC06*	*BnaMYB-CC42*	0.4011	0.0834	0.2079
*BnaMYB-CC07*	*BnaMYB-CC17*	0.3059	0.0673	0.2200
*BnaMYB-CC07*	*BnaMYB-CC19*	0.2916	0.0673	0.2308
*BnaMYB-CC09*	*BnaMYB-CC26*	0.4324	0.1823	0.4216
*BnaMYB-CC09*	*BnaMYB-CC29*	0.2448	0.1710	0.6985
*BnaMYB-CC09*	*BnaMYB-CC32*	0.4324	0.1823	0.4216
*BnaMYB-CC09*	*BnaMYB-CC35*	0.2867	0.1749	0.6100
*BnaMYB-CC10*	*BnaMYB-CC13*	0.0126	0.0150	1.1905
*BnaMYB-CC13*	*BnaMYB-CC34*	0.3190	0.0684	0.2144
*BnaMYB-CC16*	*BnaMYB-CC18*	0.1115	0.0269	0.2413
*BnaMYB-CC17*	*BnaMYB-CC19*	0.1061	0.0090	0.0848
*BnaMYB-CC20*	*BnaMYB-CC24*	0.1083	0.0099	0.0914
*BnaMYB-CC21*	*BnaMYB-CC23*	0.1280	0.0290	0.2266
*BnaMYB-CC26*	*BnaMYB-CC29*	0.2781	0.0420	0.1510
*BnaMYB-CC26*	*BnaMYB-CC32*	0.0307	0.0152	0.4951
*BnaMYB-CC26*	*BnaMYB-CC35*	0.2962	0.0394	0.1330
*BnaMYB-CC27*	*BnaMYB-CC33*	0.0809	0.0467	0.5773
*BnaMYB-CC28*	*BnaMYB-CC34*	0.0187	0.0078	0.4171
*BnaMYB-CC29*	*BnaMYB-CC32*	0.2973	0.0435	0.1463
*BnaMYB-CC29*	*BnaMYB-CC35*	0.0472	0.0073	0.1547
*BnaMYB-CC30*	*BnaMYB-CC36*	0.0779	0.0237	0.3042
*BnaMYB-CC32*	*BnaMYB-CC35*	0.3158	0.0409	0.1295
*BnaMYB-CC40*	*BnaMYB-CC42*	0.1115	0.0077	0.0691
*BnaMYB-CC43*	*BnaMYB-CC45*	0.1013	0.0485	0.4788
*BnaMYB-CC44*	*BnaMYB-CC47*	0.1372	0.0653	0.4759
*BnaMYB-CC46*	*BnaMYB-CC49*	0.1669	0.0122	0.0731

The *ka* (non-synonymous mutation rate) and *ks* (synonymous mutation rate) were calculated using the DnaSP program to better understand the effects of evolutionary constraints on *BnaMYB-CC* genes in *B. napus*. The ratios of *ka* and *ks* of two gene pairs (*BnaMYB-CC02* and *BnaMYB-CC06*; *BnaMYB-CC10* and *BnaMYB-CC13*) were higher than one, indicating that the evolutionary process was actively selected and these genes may function redundantly, resulting in fast evolution (Table 2). However, the remaining 34 gene pairs’ *ka* and *ks* rations were less than one, indicating that most *BnaMYB-CC* genes were conservative and selected in the evolutionary process, resulting in a slower evolutionary rate (Table 2).

### Gene structure and conserved motifs

**Figure 4:**
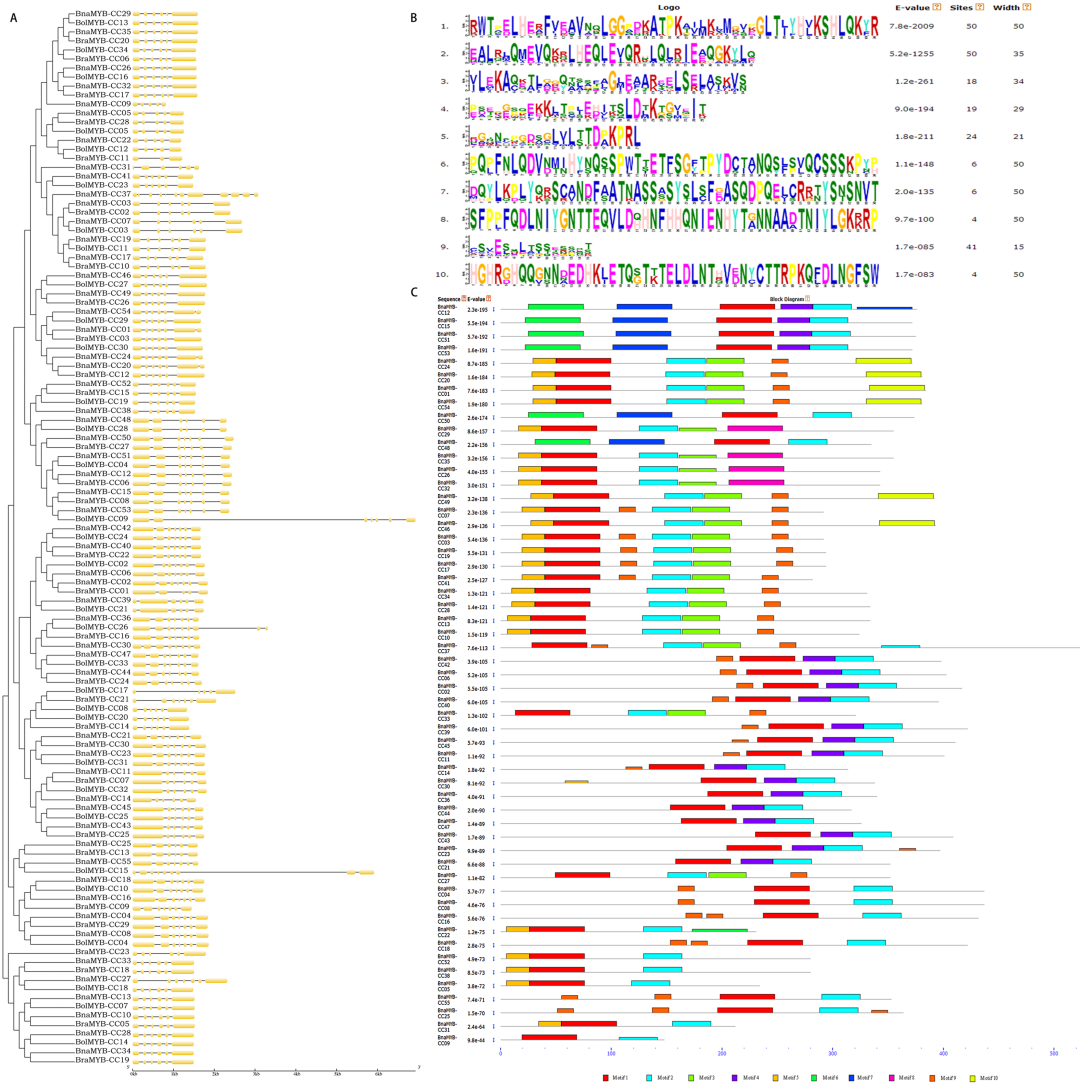
Phylogenetic analysis, exon/intron organization and conserved motifs of *MYB-CC* genes.

To better understand the diversity of MYB-CC members, the gene exon/intron structure and conserved protein motifs were investigated. All *BnaMYB-CC* genes were interrupted by several introns, and the exon/intron distribution was complicated (Fig. 4A). *BnaMYB-CC37* contained the most introns (11), while several members in group A had the fewest introns (*BnaMYB-CC05*, *BnaMYB-CC09*, and *BnaMYB-CC22* with four introns each). The members belonging to the same group showed similar exon/intron arrangements (Fig. 4A). For example, the *BnaMYB-CC* genes in Group H had five introns with similar positions and lengths, suggesting they may have resulted from gene duplication.

Forty-five pairs of homologous genes, which have the closest genetic distance, were analyzed to further investigate the variation of gene structure after allotetraplpidy. The results indicated that the genes were clustered together at the terminal level of the phylogenetic tree (Table S5). Seven pairs of homologous genes showed intron loss/gain variations, while the remaining 38 pairs had the same number of introns (Table S5). In contrast to their ancestral genes, most *BnaMYB-CC* genes had an identical exon/intron phase (Fig. 4A). It should be noted that intron acquisition events mainly occurred in group C. Specifically, *BnaMYB-CC01, BnaMYB-CC20*, and *BnaMYB-CC54* had one more intron than their homologous genes in the diploid progenitors (Table S5). The majority of the homologous pairs retained consistent number and a similar phase of exon/intron, indicating that *MYB-CC* gene structures was conserved.

Ten conserved motifs (motifs 1–10) with lengths ranging from 15–50 amino acids were identified using the MEME program (Fig. 4B). The motifs were annotated using the InterProScan program. The results showed that the MYB-like DNA-binding domain and the predicted coiled-coil domain (motif 1 and 2) were found in all BnaMYB-CCs (Fig. 4C). Moreover, similar to the exon/intron organization, the members belonging to the same group also showed similar motif composition. Motif 6 and 7 were found uniquely in the BnaMYB-CC proteins in Group D. Only the proteins of Group A contained motif 8, while motif 10 was found mainly in Group C (Fig. 4C). The other six motifs were widely distributed among the groups. For example, motif 9 was absent in Group A and Group D (Fig. 4C) indicating that there were similarities in the conserved sequences of different phylogenetic groups.

### Cis-elements in promoters of *MYB-CC* genes

**Figure 5:**
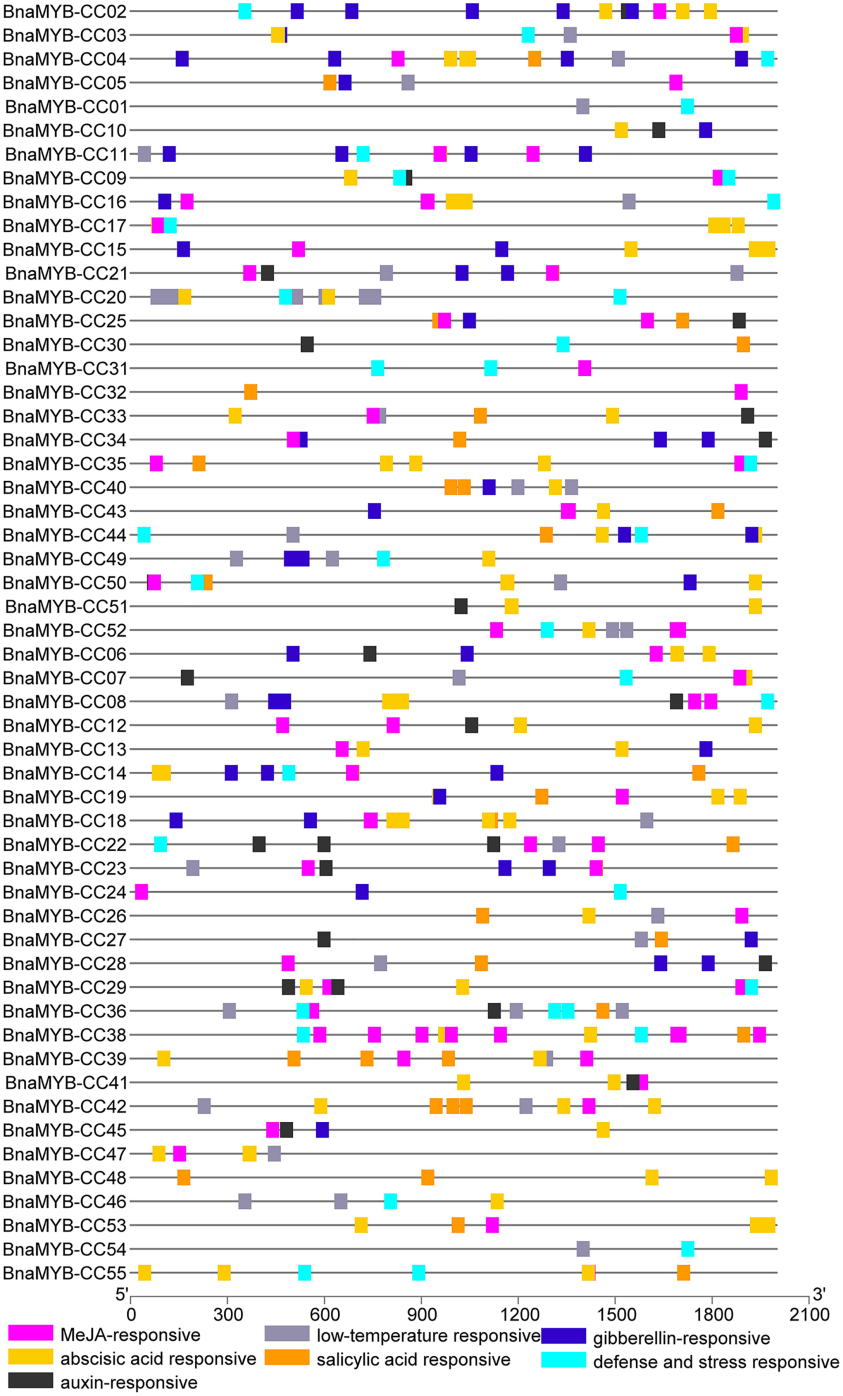
*Cis*-elements analysis of *BnaMYB-CC* genes. The 2 kb sequences upstream from the transcription start site were investigated. Different colored boxes represent different *Cis*-acting elements.

To investigate the *cis-*elements in the promoters of *MYB-CC* genes, a 2 kb sequence upstream of the start codon of each gene was extracted from the *B. napus* genome. The promoter sequences were submitted to PlantCARE. As shown in Fig. 5 and Table S6, two types of *cis-*elements (phytohormone-related and abiotic stress-related elements) were selected. Methyl jasmonate (MeJA)-responsive element (TGACG) and ABA-responsive elements (ABRE) were present in 78.1% and 74.5% of promoters of *BnaMYB-CCs*, respectively. In the promoters of 55 *BnaMYB-CCs*, 21 contained auxin-responsive elements (TGA-element, AuxRE, and AuxRR-core), 26 contained SA responsive element (TCA element), and 27 contained gibberellin-responsive elements (GARE-motif, TATC-box, and P-box). Two *cis*-lements (TC-rich repeats and LTR, related to abiotic stress) were widely distributed in *BnaMYB-CC* promoters. *BnaMYB-CC20* had seven LTRs in its promoter (Fig. 5), indicating that *BnaMYB-CC20* may play a vital role in response to low-temperature stresses. The results suggest *BnaMYB-CCs* may be involved in stress resistance and the hormone signaling pathway. It is worth noting that the *cis*-elements related to light response were found in significant quantity in the promoters of *BnaMYB-CC*. However, our work focused on the *cis*-elements related to phytohormones and thus, light responsive *cis*-elements are not shown in Fig. 5.

### Expression profiles of *MYB-CC* genes in different tissues

**Figure 6:**
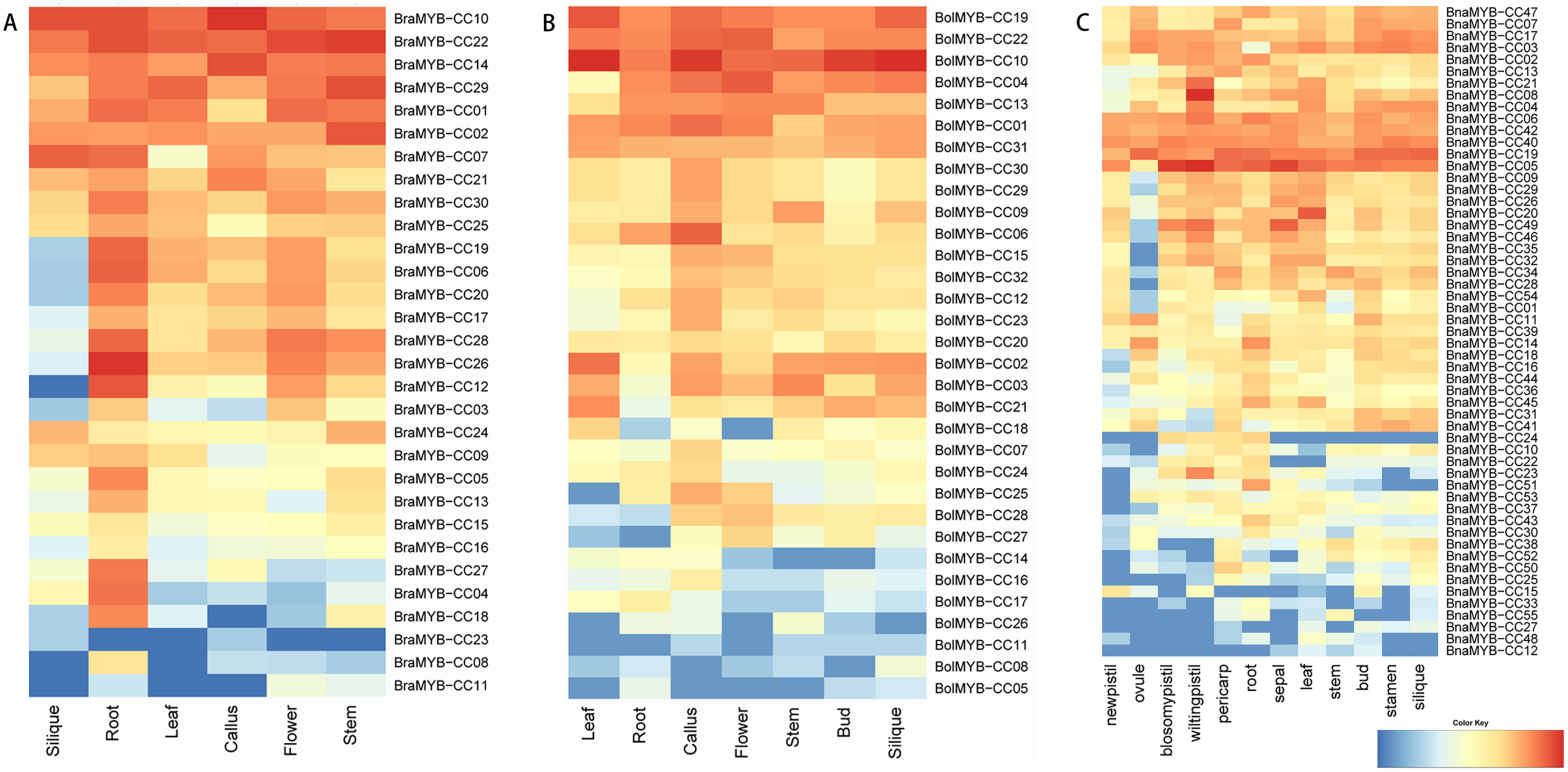
Expression patterns of *MYB-CC* genes in tissues. (A) Expression levels of 30 *BraMYB-CC* genes in six tissues of *B. rapa*. (B) Expression levels of 32 *BolMYB-CC* genes in seven tissues of *B. oleracea*. (C) Expression levels of 55 *BnaMYB-CC* genes in 12 tissues of *B. napus*.

To further understand the biological functions of *MYB-CCs*, the expression profiles of the genes in different tissues of *B. napus, B. rapa*, and *B. oleracea* were assayed based on the transcriptomic data. As shown in Fig. 6, *MYB-CC* genes were expressed widely in diverse tissues. There was a high accumulation of *BraMYB-CCs* transcripts found in the roots (Fig. 6A; Table S7). *BraMYB-CCs* in Group A (*BraMYB-CC06*, *BraMYB-CC17*, *BraMYB-CC20*, and *BraMYB-CC28*) and Group C (*BraMYB-CC03*, *BraMYB-CC12*, and *BraMYB-CC26*) were extensively expressed in tissues except for silique (Fig. 6A). The transcripts of *BolMYB-CCs* were generally found in callus (Fig. 6B; Table S7). In *B. napus*, *BnaMYB-CC05*, *BnaMYB-CC06*, *BnaMYB-CC19*, *BnaMYB-CC40, and BnaMYB-CC42* were significantly expressed in all tissues (Fig. 6C). The majority of *BnaMYB-CC* genes in Group A (*BnaMYB-CC09*, *BnaMYB-CC26*, *BnaMYB-CC29*, *BnaMYB-CC32*, and *BnaMYB-CC35*) and Group C (*BnaMYB-CC01*, *BnaMYB-CC20*, *BnaMYB-CC46*, *BnaMYB-CC49*, and *BnaMYB-CC54*) showed a low level of expression in the ovule, while their transcripts were found in other tissues (Fig. 6C). *BnaMYB-CC12*, *BnaMYB-CC15*, *BnaMYB-CC27*, *BnaMYB-CC33*, *BnaMYB-CC48*, and *BnaMYB-CC55* were expressed in fewer tissues (Fig. 6C).

### Expression analysis of *MYB-CC* genes under low Pi stresses

**Figure 7:**
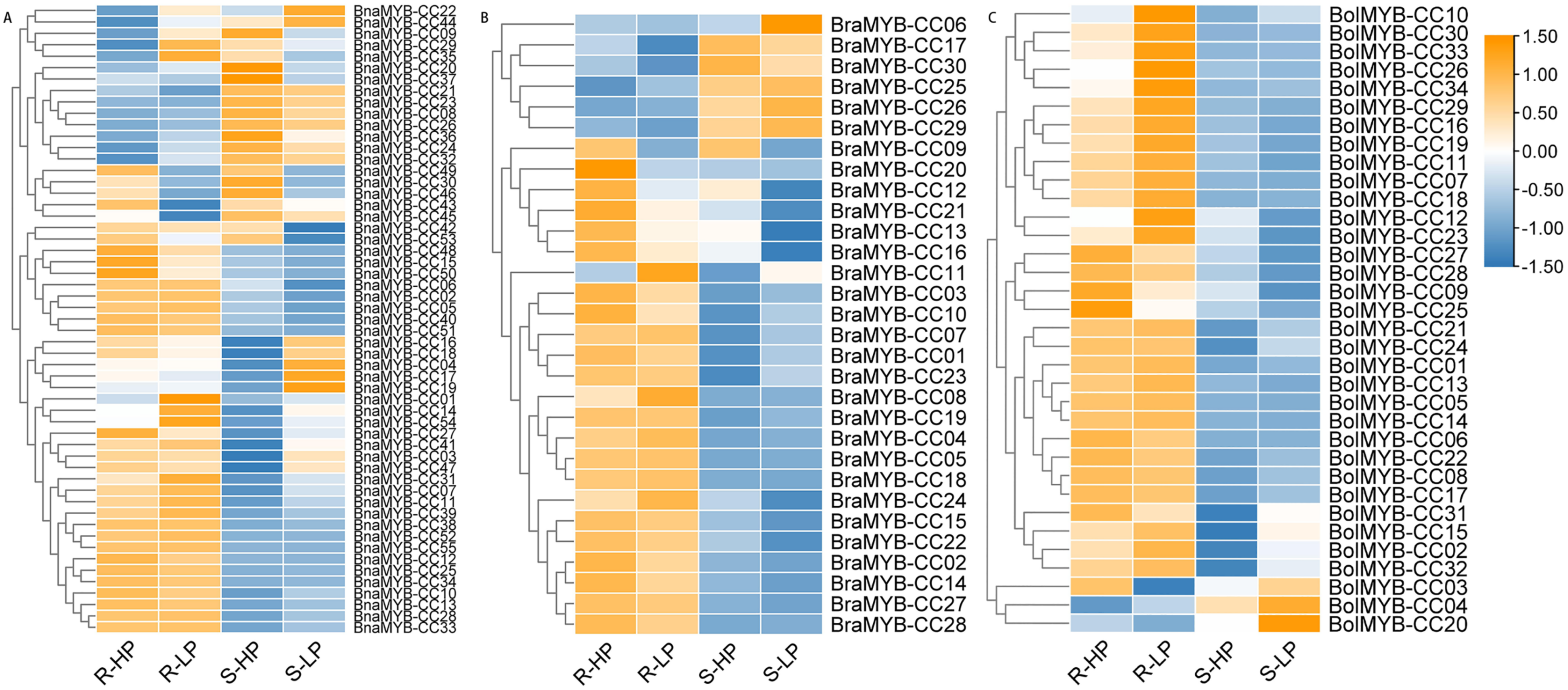
Expression patterns of *MYB-CC* genes in response to low Pi stresses. (A) Expression levels of 30 *MYB-CC* genes in *B. rapa*. (B) Expression levels of 34 *MYB-CC* genes in *B. oleracea*. (C) Expression levels of 55 *MYB-CC* genes in *B. napus*. HP, high Pi condition; LP, low Pi condition. The heat map was created using TBtools.

The expression profiles of *MYB-CC*s under low Pi stress were analyzed by RNA-Seq data to identify potential *MYB-CCs* with regulatory roles in Pi starvation responses. The original FPKM values of the transcriptome are shown in Table S8. A heatmap was constructed to display diverse expression levels in the roots and shoots under low or high Pi conditions (Fig. 7). The expression of most *MYB-CC* genes was more active in the roots than that in the shoots, especially *BraMYB-CCs* and *BolMYB-CCs* (Fig. 7). The expression patterns of several genes were altered after allopolyploidy. For example, *BraMYB-CC24*, *BolMYB-CC16*, and *BolMYB-CC31* were mainly expressed in the roots, while their orthologous genes *BnaMYB-CC44*, *BnaMYB-CC26*, and *BnaMYB-CC23* were expressed dramatically in the shoots (Fig. 7). However, other genes retained the same expression pattern after allopolyploidy, implying that the *BnaMYB-CC* genes were conserved and maintained similar functions in diploid progenitors (Fig. 7; Table S8).

Approximately half of the *BnaMYB-CCs* were induced by low Pi stress in both roots and shoots (Fig. 7). Notably, some *BnaMYB-CCs* were induced twofold or more in roots (*BnaMYB-CC01*, *BnaMYB-CC32*, and *BnaMYB-CC44*) or shoots (*BnaMYB-CC04*, *BnaMYB-CC10*, *BnaMYB-CC16*, and *BnaMYB-CC18*) under low Pi conditions (Table S8). In *B. rapa* and *B. oleracea*, approximately one-third of *BraMYB-CCs* and two-thirds of *BolMYB-CCs* genes were up-regulated under low Pi stress in roots (Fig. 7; Table S8). In *B. napus, BnaMYB-CCs* of Group D (*BnaMYB-CC12*, *BnaMYB-CC15*, *BnaMYB-CC48*, *BnaMYB-CC50*, *BnaMYB-CC51*, and *BnaMYB-CC53*) were significantly down-regulated in both roots and shoots under low Pi stress (Fig. 7C; Table S8). Seven *BnaMYB-CCs* of Group A were moderately induced by low Pi stress in roots, while their expression levels increased in shoots under high and low Pi conditions (Fig. 7; Table S8). However, *BnaMYB-CC10*, *BnaMYB-CC13*, *BnaMYB-CC27*, and *BnaMYB-CC34* of Group H were induced by low Pi stress in roots prominently. All six members of *BnaMYB-CCs* in Group H were induced by low Pi stress in shoots (Fig. 7; Table S8). The results indicate that the expression patterns were conservative for *MYB-CCs* of the same evolutionary group.

## Discussion

The MYB-CC family is a subtype within the MYB superfamily. It is contains the MYB domain and the predicted coiled-coil (CC) domain. The MYB-CC family has 15 members in *A. thaliana* (Rubio et al., 2001). PHR1, PHL1, PHL2, and PHL3 have been redundantly involved in regulating Pi starvation responses (Rubio et al., 2001; Bustos et al., 2010; Sun et al., 2016). PHL4 is one of the MYB-CC in Arabidopsis, and was regarded as an unimportant regulator in responses to Pi starvation (Wang et al., 2018). MYB-CCs have also been shown to be involved in plant growth regulation. One member of the MYB-CC Group A, APL, plays a dual role in inhibiting xylem differentiation and promoting phloem differentiation during vascular development (Bonke et al., 2003). MYR1 and MYR2 of MYB-CC Group C are redundant negative flowering time regulators under low light induction (Zhao et al., 2011). GFR of MYB-CC Group D is a low-temperature regulator of flavonoid accumulation (Petridis et al., 2016). The MYB-CC family was also mentioned in other plant species, such as 12 ZmMYB-CCs in maize (Bai et al., 2019) and 13 FvMYB-CCs in woodland strawberry (Wang et al., 2019). *MYB-CC* genes in wheat have been reported in response to drought stress (Li et al., 2019c). Additionally, BnPHR1, which is a vital regulator of low Pi responses, was isolated and identified in *B. napus* (Ren et al., 2012). The comprehensive identification and evolutionary analysis of the MYB-CC family in *B. napus* and its parental species *B. rapa* and *B. oleracea* have not been reported to date. We comprehensively analyzed the MYB-CC family in *Brassica* species and assayed their expression patterns under low Pi stress.

Gene duplication is the main driving force for gene family expansion (Taylor & Raes, 2004). *Brassica* species shared multiple paleo-polyploidy (whole-genome duplication) events with *A. thaliana*, providing primitive genetic material for biological evolution and facilitating the adaption to the changing environments (Bowers et al., 2003). Compared to the 15 *MYB-CC* genes in Arabidopsis, the number of *MYB-CC* genes in *B. napus* (55), *B. rapa* (30), and *B. oleracea* (34) increased significantly. This is due to the additional whole-genome triplication (WGT) event in the *Brassica* species after its differentiation from *A. thaliana* (Lysak et al., 2005; Town et al., 2006; Wang et al., 2011). *B. napus* is a heterotetraploid formed by hybridization between *B. rapa* and *B. oleracea*, followed by chromosome doubling (Cheung et al., 2009; Chalhoub et al., 2014; Wu et al., 2019). The segmental and tandem duplication events contribute to the gene family expansion during evolution (Cannon et al., 2004). We found that 35 *BnaMYB-CC* genes formed 36 pairs with high gene and protein sequence identity and similarity. All of these gene pairs were identified as segmental repeats, indicating that the amplification of the *MYB-CC* gene family in *B. napus* may be independent of tandem duplication. The same duplication pattern was reported in other gene families during the evolution from diploid to allotetraploid (Li et al., 2019a; Li et al., 2019b; Wang et al., 2020), and the gene families were mainly influenced by segmental duplication resulting from WGT and allopolyploidy (Li et al., 2017). Specifically, plants retain many duplicate chromosome blocks in their genomes due to polyploidization events, resulting in segmental duplication (Cannon et al., 2004). Tandem repeats are the result of unequal crossing-over between similar alleles (Achaz et al., 2000). Although no tandem duplication was found in this study, it is still an important mechanism for the expansion of gene family in plants. For instance, tandemly arrayed genes comprise more than 10% of the genes in *A. thaliana* genome (Rizzon, Ponger & Gaut, 2006).

Approximately 45 *MYB-CC* genes were predicted to exist in both *B. rapa* and *B. oleracea* as a result of the additional WGT in *Brassica* species. However, only 30 and 34 *MYB-CC* genes occurred in two diploid species and large-scale gene loss after WGT, respectively (Cheng, Wu & Wang, 2014). The *B. rapa* genome contains approximately twice the number of genes of *A. thaliana* due to genomic shrinkage and differential loss of duplicated genes after WGT events (Mun et al., 2009). It has been previously reported that 35% of genes in *Brassica* were lost by a deletion mechanism when WGT occurred (Town et al., 2006). The concentration of some gene products were changed after WGT, which may lead to an imbalance of gene dose. The relatively low retention frequency of these dose-changed genes may also explain the genetic loss after WGT (Freeling, 2008). The number of *MYB-CC* genes in *B. napus* was found to be less than the sum of its two diploid ancestors, indicating gene loss also happened after allopolyploidization. The 13 deleted genes were listed in Table S4, of which five genes belong to the A subgenome and eight belong to the C subgenome. This may be due to the rearrangement of genome sequences after hybridization (Paterson, Bowers & Chapman, 2004). In general, each of *MYB-CCs* in Arabidopsis was expected to have six homologs in *B. napus*. However, 12 *MYB-CCs* of Arabidopsis had less than six homologous genes in *B. napus*. For example, *MYR1* had only two homologs (*BnaMYB-CC46* and *BnaMYB-CC49*), which may be caused by the loss of unnecessary duplicates during evolution. The retained *MYB-CC* gene replications may play a non-redundant role in *B. napus*.

MYB-CC transcription factors are mainly involved in the responses to Pi starvation (Rubio et al., 2001; Nilsson, Muller & Nielsen, 2007; Zhou et al., 2008; Valdes-Lopez et al., 2008; Bustos et al., 2010; Ren et al., 2012; Wang et al., 2013; Sun et al., 2016; Ruan et al., 2017; Wang et al., 2018; Wang et al., 2019). Data on *MYB-CC* genes in *B. napus* are limited; however, the homologous *AtMYB-CCs* may predict the functions of *BnaMYB-CCs* in the same phylogenetic group. In Arabidopsis, *PHL2* is modulated by Pi starvation and is redundant with *PHR1* in regulating the responses to Pi starvation (Sun et al., 2016). The homologs of *PHL2* in *B. napus* (*BnaMYB-CC03*, *BnaMYB-CC07*, *BnaMYB-CC17*, and *BnaMYB-CC19*) were only slightly induced in shoots under low Pi conditions (Fig.7; Table S8), suggesting that the functions of the genes might have changed. The transcription of *AtPHR1* was not significantly regulated by Pi starvation (Rubio et al., 2001) and its homologous genes (*BnaMYB-CC02*, *BnaMYB-CC06*, *BnaMYB-CC39*, *BnaMYB-CC40*, and *BnaMYB-CC42*) in *B. napus* were not induced by low Pi stresses either (Fig.7; Table S8). The homologous genes of *AtPHR1* were also inactive at the transcriptional level under low Pi stress in other plant species (Zhou et al., 2008; Wang et al., 2019). Our results support that post-translational modification mainly regulates the activity of PHR1 (Miura et al., 2005). To date, the functions of the majority of MYB-CC genes in Arabidopsis and in other plant species are still unclear. Under low Pi stress, MYB-CC genes are mainly involved in anthocyanin biosynthesis and accumulation, Pi redistribution and homeostasis in plant shoots, and involved in modification of root system architecture and Pi uptake in plant roots. The differentiation of the expression patterns of *MYB-CC* genes from diploid to allotetraploid suggests that their role assignments in above mentioned processes could be changed. However, the functions of each *MYB-CC* gene in plant Pi homeostasis and low Pi response need to be further studied.

To further characterize the *MYB-CC* genes in responses to Pi starvation evolutionarily, we selected 38 pairs of potential orthologous genes for expression analysis. The expression levels of *BnaMYB-CCs* were significantly lower than in diploid species (Table S9). Some gene pairs with the same location had different expression patterns, whereas others with different location shared similar expression patterns (Table S9). There may be no direct correlation between the relative position of the gene and its expression patterns.

## Conclusions

This study comprehensively evaluated the identification, classification, expression and evolution analyses of *MYB-CC* gene family of *Brassica* species. A total of 30, 34, and 55 *MYB-CC* genes were identified in *B. rapa, B. oleracea*, and *B. napus*, respectively. All of the *MYB-CC* genes were divided into nine groups in the phylogenetic tree. Members of the same group have similar gene structures and conserved motifs, indicating that they could be conserved during evolution. In *Brassica*, WGT and allotetraploidy were vital for the expansion of *MYB-CC* genes. Gene loss occurred widely for a number of reasons during the evolutionary process. An analysis of gene expression under low Pi stress showed that there was no significant relationship between the relative positions of *MYB-CC* genes and their expression patterns. This work will promote the understanding of the *MYB-CC* gene family and assist further analysis of the specific functions of *MYB-CC* genes in the *Brassica* species.

## Supplemental Information

10.7717/peerj.12882/supp-1 Supplemental Information 1 MYB-CC gene family in *B. rapa*.

10.7717/peerj.12882/supp-2 Supplemental Information 2 MYB-CC gene family in *B. oleracea*.

10.7717/peerj.12882/supp-3 Supplemental Information 3 The number of MYB-CC genes in every chromosome in *B. napus*, *B. rapa* and B.oleracea.

10.7717/peerj.12882/supp-4 Supplemental Information 4 The selected differential genes in *B. napus*, *B. rapa* and *B. oleracea*.

10.7717/peerj.12882/supp-5 Supplemental Information 5 Cis-elements in the promoters of MYB-CC genes.

10.7717/peerj.12882/supp-6 Supplemental Information 6 The FPKM values of BraMYB-CC genes in RNA-Seq analysis.

10.7717/peerj.12882/supp-7 Supplemental Information 7 Intron information of MYB-CC gene pairs with the closest evolutionary relationships.

10.7717/peerj.12882/supp-8 Supplemental Information 8 The FPKM values of BnaMYB-CC genes in RNA-Seq analysis.

10.7717/peerj.12882/supp-9 Supplemental Information 9 The expression comparison of MYB-CC genes with relative position changed/maintained.

10.7717/peerj.12882/supp-10 Supplemental Information 10 RNAseq dataset of B. napus.

10.7717/peerj.12882/supp-11 Supplemental Information 11 RNAseq dataset of *B. oleracea*.

10.7717/peerj.12882/supp-12 Supplemental Information 12 RNAseq dataset of B. rapa.

## Additional Information and Declarations

### DNA Deposition

The following information was supplied regarding the deposition of DNA sequences: The raw reads from this study are available at NCBI: PRJNA739537.

### Data Availability

The following information was supplied regarding data availability: The RNA-seq data are available in the Supplemental Files and NCBI: PRJNA739537.
